# LDL-C/HDL-C Ratio Predicts Carotid Intima-Media Thickness Progression Better Than HDL-C or LDL-C Alone

**DOI:** 10.1155/2011/549137

**Published:** 2011-07-05

**Authors:** Mika Enomoto, Hisashi Adachi, Yuji Hirai, Ako Fukami, Akira Satoh, Maki Otsuka, Shun-Ichi Kumagae, Yasuki Nanjo, Kuniko Yoshikawa, Eishi Esaki, Eita Kumagai, Kinuka Ogata, Akiko Kasahara, Eri Tsukagawa, Kanako Yokoi, Kyoko Ohbu-Murayama, Tsutomu Imaizumi

**Affiliations:** ^1^Division of Cardio-Vascular Medicine, Department of Internal Medicine, Kurume University School of Medicine, Kurume 830-0011, Japan; ^2^Department of Community Medicine, Kurume University School of Medicine, Kurume 830-0011, Japan

## Abstract

High-density lipoprotein cholesterol (HDL-C) and low-density lipoprotein cholesterol (LDL-C) are strong predictors of atherosclerosis. Statin-induced changes in the ratio of LDL-C to HDL-C (LDL-C/HDL-C) predicted atherosclerosis progression better than LDL-C or HDL-C alone. However, the best predictor of subclinical atherosclerosis remains unknown. Our objective was to investigate this issue by measuring changes in carotid intima-media thickness (IMT). A total of 1,920 subjects received health examinations in 1999, and were followed up in 2007. Changes in IMT (follow-up IMT/baseline IMT × 100) were measured by ultrasonography. Our results showed that changes in IMT after eight years were significantly related to HDL-C (inversely, *P* < 0.05) and to LDL-C/HDL-C ratio (*P* < 0.05). When the LDL-C/HDL-C ratios were divided into quartiles, analysis of covariance showed that increases in the ratio were related to IMT progression (*P* < 0.05). This prospective study demonstrated the LDL-C/HDL-C ratio is a better predictor of IMT progression than HDL-C or LDL-C alone.

## 1. Introduction

A low level of high-density lipoprotein cholesterol (HDL-C) is a strong and independent predictor of cardiovascular disease [1–3]. Experimental studies have also shown that increased levels of low-density lipoprotein cholesterol (LDL-C) play a role in the development and progression of atherosclerosis [3–5]. Moreover, previous studies reported statin-induced changes in the ratio of LDL-C to HDL-C (LDL-C/HDL-C ratio) predicted atherosclerosis progression [6, 7]. It is, therefore, important to observe not only HDL-C or LDL-C alone but also their ratio. However, it is not known whether lipid levels alone or their ratio is more useful clinically for predicting the progression of subclinical atherosclerosis. 

Elevated levels of LDL-C/HDL-C or apolipoprotein (apo) B_100_/A-1 ratios were reported in patients with coronary atherosclerosis [8–10]. It has been shown that a high LDL-C/HDL-C ratio is a strong predictor of cardiovascular events [6, 11]. However, whether a high LDL-C/HDL-C ratio is a significant predictor of carotid atherosclerotic burden remains unclear due to a lack of data from a large number of subjects. Although several investigators [12, 13] reported that LDL-C/HDL-C ratio is related to IMT or carotid plaque in cross-sectional studies or in relation to baseline data in childhood, no large-scale prospective studies have been done to in adults to evaluate whether elevated levels of the LDL-C/HDL-C ratio is a more significant predictor of the progression of IMT than LDL-C or HDL-C alone. Accordingly, we measured lipid profiles and employed high-resolution ultrasonography to determine common carotid IMT at baseline in 1,920 subjects of a community-based cohort, and examined changes in IMT in a follow-up examination 8 years later.

## 2. Methods

### 2.1. Study Subjects

A periodic epidemiological survey was performed in 1999 in a small farming community in Japan (a cohort of the Seven Countries Study) on the island of Kyushu. As reported previously, the demographic backgrounds of the subjects in this area are similar to those of the Japanese general population [14]. The subjects' medical history, especially past history of cerebro-cardio vascular diseases, was ascertained in detail by a team of physicians. IMT was measured by means of high-resolution carotid ultrasonography in 1,920 subjects (794 men and 1,126 women) over the age of 40 years. Common carotid IMT was measured by duplex ultrasonography (SSA-380A, Toshiba) with a 10-MHz transducer in the sitting position. Longitudinal B-mode images at the diastolic phase of the cardiac cycle were recorded. The images were magnified and printed with a high-resolution line recorder (LSR-100A, Toshiba). IMT was measured using fine slide calipers at three levels of the lateral and medial walls one to three centimeters proximal to the carotid bifurcation. These six combined near- and far-wall measurements were averaged. In our laboratory, interobserver and intraobserver variabilities of IMT were 3.8% and 4.2%, respectively (*n* = 30).

Eight years later, we performed a follow-up examination. IMT was measured in the same manner as in the original examination. Investigators at re-examination were blinded to the participants' clinical characteristics and to their baseline IMT values. Changes in IMT were calculated as the value of follow-up IMT divided by baseline IMT, and were expressed as a percentage. Of 1,920 subjects in the original study, baseline lipid profiles were missing in 164 subjects, and follow-up IMT could not be performed in 300 subjects (183 had died, 73 refused the re-examination, 37 were lost to follow-up, and 7 had moved). In the end, complete data sets were available from 1,456 subjects, for a follow-up rate of 75.8%.

Informed consent was obtained from all subjects. The study was approved by the Kurume University Ethics Committee.

### 2.2. Study Protocol

The subjects' medical history, alcohol intake, and smoking habits were ascertained by a questionnaire. Alcohol intake and smoking habits were classified as current habitual use or not. Height and weight were measured, and body mass index (BMI) was calculated as weight (kg) divided by the square of height (m^2^) as an index of obesity. Waist circumference was measured at the level of the umbilicus in the standing position. Blood pressure (BP) was measured in the right arm twice with a mercury sphygmomanometer after subjects had rested in a supine position for more than 5 minutes. The second BP with the fifth-phase diastolic pressure was used for analysis.

Blood was drawn from the antecubital vein for determinations of glycosylated hemoglobin A_1c_ (HbA_1c_), lipids profiles (total cholesterol, LDL-C, HDL-C, triglycerides (TG), and remnant-like lipoprotein particle cholesterol (RLP-C), and free fatty acid (FFA)) in the morning after 12-hour fast. Fasting blood samples were centrifuged immediately after collection. Serum total cholesterol, LDL-C, HDL-C, TG, FFA, and creatinine were measured by enzymatic assay method, and RLP-C was measured by an immunoseparation technique using an immunoaffinity gel containing monoclonal antibodies to human apo B_100_ and apo A-1 [15]. Non-HDL-C was calculated by subtracting HDL-C from total cholesterol. Similarly, we evaluated ratios of proatherogenic lipoprotein measurements, including total cholesterol to HDL-C (TC/HDL-C), LDL-C to HDL-C (LDL-C/HDL-C), TG to LDL-C (TG/LDL-C), and TG to HDL-C (TG/HDL-C). HbA_1c_ was measured by ion-exchange high-performance liquid chromatography. All blood chemistry analyses were performed at a commercial laboratory (The Kyodo Igaku Laboratory, Fukuoka, Japan).

### 2.3. Statistical Analyses

Results were presented as mean ± standard deviation (SD). Because of skewed distributions, TG, RLP-C, and FFA were log-transformed before data analyses; mean values, and upper and lower 95% confidence limits, were exponentiated and presented as geometric mean ± SD, where the SD was approximated as the difference between the exponentiated confidence limits divided by 3.92, the value of SD in a 95% confidence interval for normally distributed data. Sex, smoking habits, alcohol intake, hypertensive medication, diabetic medication, and hyperlipidemic medication were used as dummy variables.

In order to investigate factors responsible for changes in IMT after 8 years, multiple linear regression analyses were performed with age, sex, and baseline IMT. The mean changes in IMT levels stratified by quartiles of LDL-C/HDL-C ratio were compared using analysis of covariance (ANCOVA) adjusted for age, sex, baseline IMT, and lipid lowering medications. Since subjects with a greater IMT at baseline may exhibit increased changes in IMT, further analysis was performed using the subjects with an IMT less than 1.1 mm at baseline. This subanalysis used the same ANCOVA analysis as described above.

Statistical significance was defined as *P* < 0.05. All analyses were performed with the use of the SAS system (SAS Institute Inc., Software 9.2, Cary, NC, USA).

## 3. Results

Demographic data for the 1,920 subjects at baseline in 1999 are presented in Table 1. As is apparent from the table, the enrolled subjects had almost normal mean values of risk factors and other variables.  Table 2 shows the results of univariable analysis performed for correlates of IMT using multiple linear regression analysis adjusted for age and sex. At baseline, there were significant cross-sectional relationships between IMT and systolic or diastolic BPs (*P* < 0.001), BMI (*P* < 0.001), waist circumference (*P* < 0.05), total cholesterol (*P* < 0.05), HDL-C (*P* < 0.001; inversely), LDL-C (*P* < 0.001), total/HDL-C ratio (*P* < 0.001), LDL-C/HDL-C ratio (*P* < 0.001), TG/HDL-C ratio (*P* < 0.01), RLP-C (*P* < 0.05), and HbA_1c_ (*P* < 0.01). After adjusting for age, sex, BMI, and smoking habits, multiple linear regression analysis showed significant relationships between IMT and total cholesterol (*P* < 0.05), HDL-C (*P* < 0.001; inversely), LDL-C (*P* < 0.001), LDL-C/HDL-C ratio (*P* < 0.0001), TG/HDL-C ratio (*P* < 0.01), and RLP-C (*P* < 0.05). 

In Table 3, multiple linear regression analysis revealed that baseline systolic BP (*P* < 0.01), BMI (*P* < 0.05), waist circumference (*P* < 0.01), HDL-C (*P* < 0.05; inversely), TC/HDL-C ratio (*P* < 0.05), LDL-C/HDL-C ratio (*P* < 0.05), and TG/LDL-C ratio (*P* < 0.05; inversely) were significant predictors of IMT progression after 8 years, after adjustments for age, sex, and baseline IMT. LDL-C/HDL-C ratio in particular was more closely associated with outcome than any of the other individual proatherogenic lipoprotein parameters.

**Figure 1:**
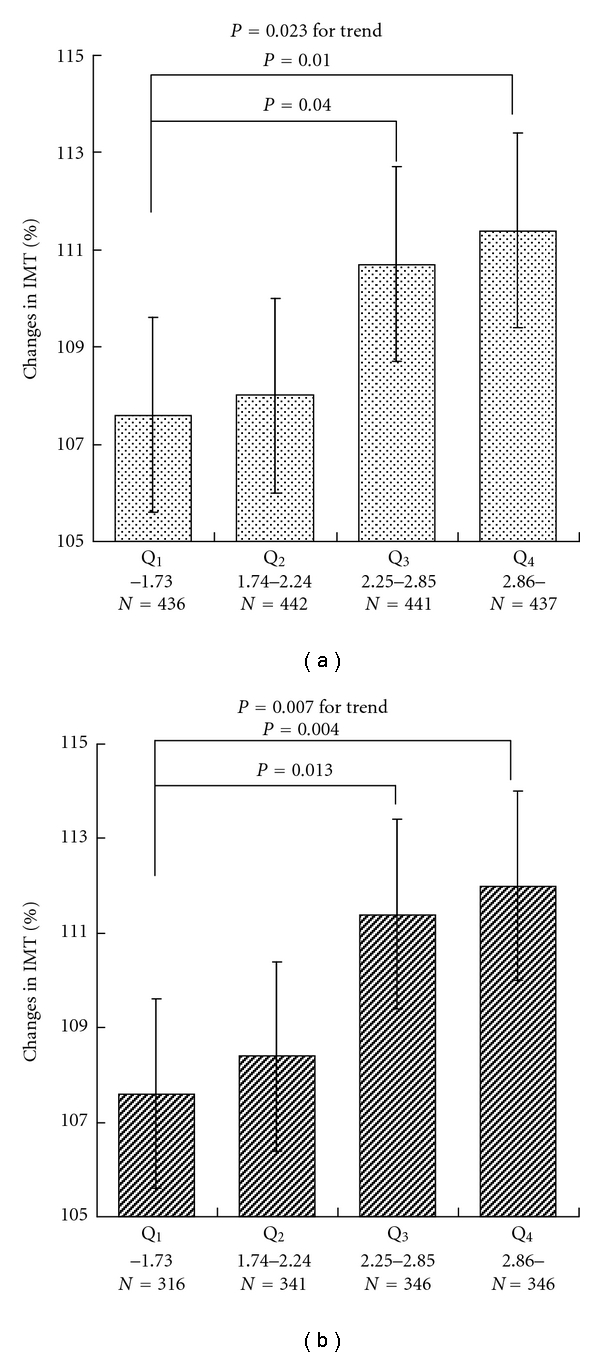
Relationship between LDL-C/HDL-C ratio and changes in IMT. (a) Data from 1,456 subjects was analyzed by ANCOVA adjusted for age, sex, baseline IMT, and lipids lowering medication. (b) Data from 1,349 subjects with less than 1.1 mm IMT at baseline was analyzed by ANCOVA adjusted for age, sex, baseline IMT, and lipid lowering medications.

Figure 1(a) shows mean changes in IMT levels stratified by quartiles of LDL-C/HDL-C ratio, compared by ANCOVA adjusted for age, sex, baseline IMT, and lipid lowering medications. Increased LDL-C/HDL-C ratios were related to IMT progression (*P* = 0.007 for trend). Further analysis was performed using the subjects with an IMT less than 1.1 mm at baseline (Figure 1(b)). This subanalysis also showed a significant linear trend between LDL-C/HDL-C ratio and IMT progression.

In analyses using the receiver operating characteristic (ROC) curve, an LDL-C to HDL-C ratio of 2.3 (area under curve 0.552) showed the strongest association with IMT progression (80.3% sensitivity and 79.3% specificity, data not shown).

## 4. Discussion

This is the first report of a general population or large-scale epidemiological study focusing on the relationship between LDL-C/HDL-C ratio and IMT progression.

### 4.1. Methodological Considerations

In this study, we evaluated changes in IMT by high-resolution carotid ultrasonography but did not measure plaques. It may have been preferable to measure changes in plaques rather than IMT. However, it is very difficult to accurately estimate the changes in plaques because of their complex morphology. In contrast, the evaluation of IMT is relatively simple and accurate. Furthermore, IMT, as an indicator of subclinical atherosclerosis, has been shown to be a strong risk factor for cardiovascular events [16]. Measurement of IMT could help to identify asymptomatic subjects with subclinical atherosclerosis who would benefit from aggressive preventive measures.

With regard to composite variables that include proatherogenic lipoprotein measurements, data for direct comparisons are limited to studies based on the general population [17, 18]. In the present study, some of the ratios and the single measurements such as LDL-C, HDL-C, and RLP-C were included in study variables. Although other population-based studies [17, 18] included antiatherogenic lipoprotein fraction ratios such as apo B/A-I, their main target endpoint was cardiovascular events. Our objective was to determine the best predictor of progression of subclinical atherosclerosis, and we used changes in IMT as a surrogate marker for atherosclerosis. As shown in Figure 1(b), since the subjects with thicker IMT tend to exhibit relatively large changes in IMT, a further analysis was performed using the subjects with less than 1.1 mm IMT at baseline. This type of careful subanalysis may help us to better understand the significance of changes in IMT.

### 4.2. Predictors for Progression of IMT

A significant association between IMT and proatherogenic lipoprotein measurements was shown in Table 2. Even in this cross-sectional study, we found LDL-C/HDL-C ratio was the strongest predictor. We observed the same result in our prospective study, as shown in Table 3. Because age, male gender, and baseline IMT are well-known significant factors for the progression of IMT, we adjusted for them to determine factors for progression of IMT. However, the present study showed that the absolute differences (0.03 mm versus 0.04 mm) and the percent differences (104.0% versus 105.9%) between the IMT at baseline and during follow-up period did not differ significantly between the two genders. Therefore, we performed an additional analysis using 1,349 subjects with an IMT less than 1.1 mm at baseline (Figure 1(b)). The results of our multiple linear regression analysis showed that the LDL-C/HDL-C ratio was the strongest predictor for IMT progression (*β* = 1.55453, *P* < 0.05). Although one prospective study [19] reported the predictive utility of LDL/HDL ratio for carotid IMT in subjects using childhood levels, ours is the first large prospective study to confirm this result in a community-based cohort without apparent cerebro-cardiovascular diseases.

Our data may suggest that elevated LDL/HDL ratio is not just a marker of atherosclerosis but may play a causal role in the pathogenesis of human IMT progression. Another interesting issue to be clarified is the cut-off values of LDL-C/HDL-C for predicting IMT progression. One Japanese investigator [20] has suggested the target index for regression of atherosclerosis should be less than 2.0 for primary prevention and less than 1.5 for secondary prevention. Based on the ROC curves, the cut-off point was 2.3 in this study. The detailed analysis stratified by LDL-C/HDL-C quartiles (Figures 1(a) and 1(b)) may confirm this cut-off value.

### 4.3. Study Limitations

This study had some limitations. First, the lack of biochemical measurements and medication data in the followup is a major limitation. Second, we did not measure total plaque area. Third, we only examined subjects older than 40 years old. It would be interesting to determine whether LDL-C/HDL-C ratio is a predictor of progression of IMT of the carotid artery in subjects younger than 40 years of age. Fourth, several pharmacological interventions have beneficial effects on prevention of the progression of IMT [21, 22]. However, because we have no data on medications taken after the baseline examination, we cannot comment on the question of whether such interventions have any beneficial effects on IMT progression. Further studies are needed to address this issue.

In conclusion, to our knowledge, this is the first epidemiological report in a community cohort to show that LDL-C/HDL-C ratio is a better predictor of carotid IMT progression than HDL-C or LDL-C alone.

## Figures and Tables

**Table 1:** Characteristics of subjects at baseline in 1999.

	Men	Women	Total
N	794	1126	1920
Age, years	63.6 ± 11.0	62.1 ± 11.0	62.7 ± 11.0
Systolic blood pressure, mmHg	135.7 ± 21.6	131.7 ± 21.4	133.4 ± 21.6
Diastolic blood pressure, mmHg	81.0 ± 13.1*	76.9 ± 11.4	78.6 ± 12.3
Body mass index, kg/m^2^	23.2 ± 3.0	23.0 ± 3.2	23.1 ± 3.1
Waist, cm	81.6 ± 8.6	73.8 ± 8.4	77.0 ± 9.3
IMT, mm	0.75 ± 0.22**	0.67 ± 0.18	0.70 ± 0.20
Absolute difference of IMT, mm	0.03 ± 0.02	0.04 ± 0.01	0.04 ± 0.01
Total cholesterol, mg/dL	188.8 ± 32.8	207.4 ± 33.8	199.8 ± 34.6
HDL-C, mg/dL	51.6 ± 15.4	57.9 ± 14.3	55.8 ± 14.0
LDL-C, mg/dL	118.1 ± 31.2	128.5 ± 30.3	124.2 ± 31.0
Triglycerides^†^, mg/dL	105.6 ± 2.7**	82.8 ± 2.4	98.5 ± 2.6
Non-HDL-C, mg/dL	136.4 ± 33.2**	149.3 ± 33.8	144.0 ± 34.2
LDL-C/HDL-C ratio	2.4 ± 0.9**	2.3 ± 0.8	2.4 ± 0.8
Total cholesterol/HDL-C ratio	3.82 ± 1.14**	3.74 ± 0.99	3.77 ± 1.06
Triglycerides/HDL-C ratio^†^	2.07 ± 0.05**	1.64 ± 0.04	1.80 ± 0.05
Triglycerides/LDL-C ratio^†^	0.92 ± 0.02**	0.74 ± 0.02	0.81 ± 0.02
Free fatty acid^†^, mEq/L	0.51 ± 0.01	0.56 ± 0.01	0.53 ± 0.01
RLP-C^†^, mg/dL	3.52 ± 0.09	3.47 ± 0.09	3.49 ± 0.09
HbA1c, %	5.2 ± 0.8	5.2 ± 0.7	5.2 ± 0.8
Smoking, %	38.8	2.0	17.2
Alcohol intake, %	48.9	3.2	22.1
Hypertensive medication, %	20.5	19.0	19.6
Diabetic medication, %	3.9	2.6	3.1
Lipids lowering medication, %	2.4	6.4	4.7

*P < 0.05, **P < 0.001, ^†^Log-transformed values were used in analyses. IMT: intima-media thickness; HDL-C: high-density lipoprotein cholesterol; LDL-C: low-density lipoprotein cholesterol; RLP-C: remnant-like particle cholesterol.

**Table 2:** Association between IMT and variables at baseline in multiple linear regression analysis adjusted for age and sex.

Variables	*β*	Standard error	Probability
Systolic blood pressure	0.00158	0.0002	<0.01
Diastolic blood pressure	0.00145	0.0003	<0.01
Body mass index	0.00444	0.0013	0.01
Waist	0.00119	0.0005	0.01
Total cholesterol	0.00028	0.0001	0.02
HDL-C	−0.00127	0.0003	<0.01
LDL-C	0.00053	0.0001	<0.01
Triglycerides*	0.00006	0.0001	0.23
Non-HDL-C	0.00049	0.0001	<0.01
LDL-C/HDL-C ratio	0.02930	0.0048	<0.01
Total cholesterol/HDL-C	0.02083	0.0037	<0.01
ratio			
Triglycerides/HDL-C ratio*	0.02034	0.0062	<0.01
Triglycerides/LDL-C ratio*	0.00237	0.0078	0.76
Free fatty acid	0.00313	0.0075	0.68
RLP-C	0.01832	0.0073	0.01
HbA1c	0.01688	0.0052	<0.01
Smoking	0.00632	0.0120	0.60
Alcohol intake	−0.02119	0.0114	0.06

*Log-transformed were used for triglycerides, triglyceride/HDL-C ratio, triglyceride/LDL-C ratio, free fatty acid, and RLP-C concentration. HDL-C: high-density lipoprotein cholesterol; LDL-C: low-density lipoprotein cholesterol; RLP-C: remnant-like particle cholesterol.

**Table 3:** Relationship between 8-year changes in IMT and variables in multiple linear regression analysis adjusted for age, sex, and baseline IMT.

Variables	*β*	Standard error	Probability
Systolic blood pressure	0.07764	0.0278	0.01
Diastolic blood pressure	0.01312	0.0464	0.78
Body mass index	0.39651	0.1681	0.02
Waist	0.16325	0.0627	0.01
Total cholesterol	0.01204	0.0155	0.44
HDL-C	−0.08175	0.0380	0.03
LDL-C	0.03103	0.0175	0.08
Triglycerides*	−0.00088	0.0060	0.88
Non-HDL-C	0.02595	0.0156	0.10
LDL-C/HDL-C ratio	1.55453	0.6466	0.02
Total cholesterol/HDL-C	1.06553	0.4974	0.03
ratio			
Triglycerides/HDL-C ratio*	0.80106	0.7886	0.31
Triglycerides/LDL-C ratio*	−1.32504	0.9724	0.03
Free fatty acid*	0.52784	1.0439	0.61
RLP-C*	0.23376	0.8359	0.75
HbA1c	0.55307	0.7430	0.46
Smoking	−0.94243	1.6585	0.57
Alcohol intake	−0.55662	1.5202	0.71

*Log-transformed were used for triglycerides, triglyceride/HDL-C ratio, triglyceride/LDL-C ratio, free fatty acid, and RLP-C concentration. HDL-C: high-density lipoprotein cholesterol; LDL-C: low-density lipoprotein cholesterol; RLP-C: remnant-like particle cholesterol.
